# Resting‐state functional magnetic resonance imaging versus task‐based activity for language mapping and correlation with perioperative cortical mapping

**DOI:** 10.1002/brb3.1362

**Published:** 2019-09-30

**Authors:** Jean‐Michel Lemée, David Hassanein Berro, Florian Bernard, Eva Chinier, Louis‐Marie Leiber, Philippe Menei, Aram Ter Minassian

**Affiliations:** ^1^ Department of Neurosurgery University Hospital of Angers Angers France; ^2^ INSERM CRCINA Équipe 17, Bâtiment IRIS Angers France; ^3^ Department of Neurosurgery University Hospital of Caen Caen France; ^4^ Angers Medical Faculty Anatomy Laboratory Angers France; ^5^ Department of Physical Medicine and Rehabilitation University Hospital of Angers Nantes France; ^6^ Department of Radiology University Hospital of Angers Angers France; ^7^ Department of Anesthesiology University Hospital of Angers Angers France; ^8^ LARIS EA 7315, Image Signal et Sciences du Vivant Angers Teaching Hospital Angers France

**Keywords:** brain surgery, brain tumor, fMRI, language, rest

## Abstract

**Introduction:**

Preoperative language mapping using functional magnetic resonance imaging (fMRI) aims to identify eloquent areas in the vicinity of surgically resectable brain lesions. fMRI methodology relies on the blood‐oxygen‐level‐dependent (BOLD) analysis to identify brain language areas. Task‐based fMRI studies the BOLD signal increase in brain areas during a language task to identify brain language areas, which requires patients' cooperation, whereas resting‐state fMRI (rsfMRI) allows identification of functional networks without performing any explicit task through the analysis of the synchronicity of spontaneous BOLD signal oscillation between brain areas. The aim of this study was to compare preoperative language mapping using rsfMRI and task fMRI to cortical mapping (CM) during awake craniotomies.

**Methods:**

Fifty adult patients surgically treated for a brain lesion were enrolled. All patients had a presurgical language mapping with both task fMRI and rsfMRI. Identified language networks were compared to perioperative language mapping using electric cortical stimulation.

**Results:**

Resting‐state fMRI was able to detect brain language areas during CM with a sensitivity of 100% compared to 65.6% with task fMRI. However, we were not able to perform a specificity analysis and compare task‐based and rest fMRI with our perioperative setting in the current study. In second‐order analysis, task fMRI imaging included main nodes of the SN and main areas involved in semantics were identified in rsfMRI.

**Conclusion:**

Resting‐state fMRI for presurgical language mapping is easy to implement, allowing the identification of functional brain language network with a greater sensitivity than task‐based fMRI, at the cost of some precautions and a lower specificity. Further study is required to compare both the sensitivity and the specificity of the two methods and to evaluate the clinical value of rsfMRI as an alternative tool for the presurgical identification of brain language areas.

## Graphical abstract

We compared task‐based and resting‐state fMRI for the identification of language network in patient surgically treated for a brain tumor. fMRI data were then compared to the standard, the electric cortical stimulation. Resting‐state fMRI was able to detect brain language areas during CM with a sensitivity of 100% compared to 64% with task fMRI.

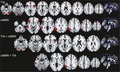

## 1 INTRODUCTION

Brain tumors represents 1.4% of tumors in adults and accounts for 2.3% of cancer‐related deaths (Bondy et al., 2008; Smedby, Brandt, Bäcklund, & Blomqvist, 2009). The goal of brain tumor surgery is a maximal resection of the tumor while minimizing the risk of postoperative deficit by sparing eloquent functional brain areas. For the brain tumors located in the vicinity of eloquent brain areas, such as motor and language areas, the identification of eloquent brain areas is essential to neurosurgical decision‐making to preserve neurological function. Cortical mapping (CM) by intraoperative direct electric stimulation during awake surgery is considered to be the gold standard for eloquent brain area identification (Ojemann, Ojemann, Lettich, & Berger, 2008).

Furthermore, the preoperative identification of eloquent brain areas through functional MRI (fMRI) is also used for the assessment of surgical risk, surgical planning, and to further guide intraoperative CM as well as other modalities like high‐density electroencephalogram, and magnetoencephalography. fMRI is performed by contrasting brain oxygen‐level‐dependent (BOLD) images between task‐based fMRI and baseline periods. The BOLD signal from each period is then compared from each other to identify the brain areas activated during the task. Task fMRI imaging has been widely used for surgical planning of brain tumors in the vicinity of eloquent brain areas (Bailey et al., 2015; FitzGerald et al., 1997; Mahdavi et al., 2015; Petrella et al., 2006; Roux et al., 2003; Wood et al., 2011). However, there are some limitations since this task‐based fMRI paradigm relies heavily on task performance, excluding a number of patients because of a preoperative cognitive or physical impairment or because of their young age.

RsfMRI identifies brain areas with a synchronous spontaneous low‐frequency oscillations of fMRI signal over time, usually below 0.1 Hz. Brain areas with a spontaneous synchronous oscillation of their BOLD signal are considered to belong to the same resting‐state functional network (RSN) (Biswal, Yetkin, Haughton, & Hyde, 1995; Fox et al., 2005). Importantly, the correlation structure of RSNs reflects the neuroanatomical substrate of task‐induced activity (Fox et al., 2005; Mitchell et al., 2013). Among other networks, RSN corresponding to the language network has been successfully identified in adults at rest (Cordes et al., 2000; Mitchell et al., 2013; Sair et al., 2016; Ter Minassian et al., 2014; Tie et al., 2014). Functional mapping using resting‐state fMRI (rsfMRI) and spatial independent component analysis (sICA) has already been used to identify eloquent brain areas and overcome the limitations of task‐based fMRI for presurgical planning (Hart, Price, & Suckling, 2016; Shimony et al., 2009; Tie et al., 2014).

The aim of this study was to compare the effectiveness of preoperative language mapping using rsfMRI and task‐based fMRI to the perioperative cortical mapping during awake craniotomies in adults.

## 2 MATERIAL AND METHOD

### 2.1 Participants

This is a monocentric prospective study including adult patient with a brain lesion treated in the Department of Neurosurgery of the University Hospital of Angers that underwent a preoperative fMRI language mapping with both rsfMRI and task fMRI as well as a perioperative CM of eloquent brain language areas in awake condition. This study was approved by the Local Ethics Committee (Comité de protection des personnes, CPP Ouest II, Angers, France, authorization date: November 15, 2012). All subjects gave their written, informed consent prior to their enrollment in this study.

For a better homogeneity of the fMRI acquisitions, the beginning of inclusion was set to October 1, 2014, date of the commissioning of the 3 Tesla MRI in our hospital. All patients were French native speakers, operated in awake surgery condition of a brain lesion, with a preoperative fMRI language mapping and a perioperative motor and language cortical mapping. Exclusion criteria were severe mental retardation, age <18 years, a preoperative language deficit making cortical mapping impossible and a quality control of fMRI data showing unusable data, for example, with head movements ≥3 mm in one of the axes during their acquisition. Fifty patients identified in accordance with inclusion criterion were included in this study. Details of the population are presented in Table 1.

**Table 1:** Characteristics of the population

Patient	Sex	Age (years)	Lesion side	Lesion location	Lesion histology and WHO grade	Language disturbance	Anxiety score	Success score
1	F	54	L	Precentral gyrus	GB	Mild	7.7	4.9
2	M	18	L	Superior frontal gyrus	DNET	No	2.7	8.9
3	F	59	L	Superior temporal gyrus	Lung adenocarcinoma metastasis	Mild	8.2	7.2
4	F	47	L	Fusiform gyrus	XA II	No	1.5	7.9
5	F	51	L	Inferior frontal gyrus	GB	No	1.3	8.2
6	M	64	L	Precentral gyrus	GB	No	1.6	4.6
7	M	35	L	Precentral gyrus	AA III	Mild	0.3	5.2
8	F	68	L	Hippocampus	GB	No	10	5.1
9	M	63	L	Middle temporal gyrus	GB	No	1.9	7.7
10	M	34	R	Superior frontal gyrus	OA III	No	4.8	5.8
11	F	29	L	Superior frontal gyrus	OA II	No	3.2	8.2
12	F	53	L	Fronto‐insular	OA III	No	0.0	5.9
13	M	36	L	Middle frontal gyrus	OA II	No	6.1	4.5
14	M	48	L	Precentral gyrus	OA III	No	6.5	8.0
15	F	60	L	SMA	GB	No	0.0	5.2
16	F	42	L	Superior frontal gyrus	OD III	No	2.4	8.0
17	M	22	L	Temporo‐insular	GG	No	7.6	7.6
18	M	67	L	Angular gyrus	GB	Mild	5.7	10
19	F	58	L	Superior parietal lobule	PA	No	NA	NA
20	M	49	L	Precentral gyrus	OA III	Mild	2.6	6.9
21	M	42	L	Inferior frontal gyrus	GB	No	0.8	6.8
22	M	30	L	Inferior temporal gyrus	OA III	No	1.8	7.0
23	M	65	L	Angular gyrus	Lung adenocarcinoma metastasis	No	3.4	4.4
24	M	52	R	Superior frontal gyrus	GS	No	2.2	9.6
25	F	69	R	Fronto‐temporo‐insular	GB	Mild	5.3	5.2
26	F	39	L	Lingual gyrus	AB	No	3.0	6.2
27	M	75	R	Middle frontal gyrus	OA III	No	5.6	3.9
28	M	58	L	Inferior frontal gyrus	Radionecrosis	No	0.8	6.6
29	M	55	L	Parahippocampal gyrus	GB	Mild	0.0	7.1
30	F	66	L	Superior frontal gyrus	GB	No	3.8	3.8
31	M	64	L	Lingual gyrus	GB	No	0.0	6.3
32	M	57	L	Parahippocampal gyrus	Cavernoma	No	4.9	6.9
33	M	47	L	Superior frontal gyrus	OD III	No	4.9	6.9
34	M	50	L	Thalamic	GB	No	0.3	7.6
35	M	52	L	Fronto‐insular	AA III	No	3.1	6.6
36	M	62	L	Parietal	GB	No	2.5	2.5
37	M	62	L	Angular gyrus	GB	Mild	1.3	4.3
38	M	50	L	Fusiform gyrus	GB	No	0.0	8.5
39	F	45	L	Inferior temporal gyrus	PA	No	0.0	8.1
40	F	51	L	Middle frontal gyrus	GB	Mild	4.3	4.4
41	M	24	L	Superior frontal gyrus	OA II	No	3.5	7.2
42	M	41	L	Precentral gyrus	OA II	No	5.0	5.3
43	M	39	L	Operculum	GB	Severe	6.5	4.6
44	M	47	L	Middle temporal gyrus	Cavernoma	No	6.0	5.0
45	M	40	L	Supramarginal gyrus	Cavernoma	No	5.0	8.3
46	F	35	L	Angular gyrus	Arteriovenous malformation	No	2.0	7.1
47	M	46	R	Angular gyrus	GB	No	5.3	3.2
48	M	56	L	Middle frontal gyrus	GB	No	4.2	8.0
49	M	69	R	Occipital	AA III	No	3.0	6.0
50	M	34	L	Supramarginal gyrus	GB	Mild	5.3	6.2

Abbreviations: AA, anaplastic astrocytoma; AB, astroblastoma; DNET, dysembryoplastic neuroectodermal tumor; GB, glioblastoma; GG, ganglioglioma; GS, gliosarcoma; OA, oligoastrocytoma; OD, oligodendroglioma; PA, pilocytic astrocytoma; SMA, supplementary motor area; XA, xanthoastrocytoma.

### 2.2 fMRI data acquisition

All datasets were acquired on a 3.0 Tesla MR Scanner (Magnetom^®^ Skyra Medical Systems™). During image acquisition, patients laid supine with the head immobilized by foam pads and straps, with earphones, and kept in darkness. Patients watched a black screen with a red fixation cross in the center through a prism.

Echo planar imaging (EPI) sequence was used for each fMRI with the following parameters TR = 2,280 ms, TE = 30 ms, flip angle  =  90°, 42 axial interleaved slice of 4 mm slice thickness, in‐plane matrix  =  64 × 64 with a field of view  = 168 × 187 mm, yielding a voxel size of 3 × 3 × 4 mm^3^, covering the whole brain including the cerebellum. During task fMRI, we acquired 270 functional volumes per session over two sessions, and for rsfMRI, we acquired 270 functional volumes over one session.

A T1‐weighted anatomical three‐dimensional dataset was also obtained, covering the whole brain to coregister and normalize EPI images, with the following parameters: 192 contiguous sagittal slices, in‐plane matrix 256 × 256, yielding a voxel size of 1 × 1 × 1 mm^3^.

### 2.3 Experimental paradigm

After completing the Edinburgh Handedness Inventory (EHI) Score (Oldfield, 1971), each patient underwent the three consecutive fMRI sessions: one rsfMRI and then two task fMRI sessions.

For rsfMRI, subjects were instructed to keep their eyes open, to fix a red cross on the screen and relax. For task fMRI acquisition, the paradigm was implemented in block designs with two conditions of sixteen seconds each: (a) During sentence generation (SG) periods, patients were asked to covertly generate short sentences semantically linked to a word heard in the earphones every four seconds and (b) for reference tone listening (TL) periods, patients at rest listened to two alternating monotonous tones every four seconds. This latter condition represented the baseline condition. Word and tones were presented using E‐Prime software (Psychology Software Tools). Before applying to patients this modified word verb matching task, we controlled his/her ability to generate robust linguistic activation in healthy volunteers (Figure S1 and Table S1).

Beforehand, all subjects received detailed instruction and were trained to perform the task overtly and then covertly. Before fMRI acquisitions, the subjects were asked to grade their anxiety score and after acquisition their estimated performance for the task fMRI. The visual analog scales were converted in a value on a scale from 0 to 10. These results were compared to data from 33 healthy volunteers that underwent the same fMRI protocol and enrolled in a previous study and were used to compare clinical data, anxiety, and success scores after fMRI acquisitions (Dinomais et al., 2016). All patients enrolled did not have language impairment at the moment of the fMRI acquisition and during the surgical procedure.

### 2.4 Analysis of imaging data

The first three acquisition volumes in each functional series were discarded, to allow the longitudinal magnetization to stabilize.

Preprocessing was carried out using SPM8 (Wellcome Department of Imaging Neuroscience, University College, London, UK, http://www.fil.ion.ucl.ac.uk/spm) running under MATLAB (The MathWorks). Each patient's native space images were corrected for time delays between slices. Then, all images were realigned to the first volume of the first session and unwrapped to correct head movement and susceptibility distortions. The three‐dimensional dataset was segmented in native space, using the VBM 8.0 toolbox for SPM^®^ and coregistered to the mean functional image using gray matter segmentation as a reference image. The coregistered gray matter segmentation was then used to spatially normalize data into a standard template provided by the Montreal Neurological Institute (MNI template) with a final resolution of 3 × 3 × 3 mm. Finally, the images were spatially smoothed with a 6‐mm kernel of full width at half‐maximum.

For task fMRI analysis, the two conditions were the two successive epochs of a trial: TL and SG. A generalized linear model approach was used with regressors corresponding to each of the two conditions SG and TL convolved with a model of canonical hemodynamic response incorporated in the SPM8 package. Each individual time series of the preprocessed datasets was then analyzed by voxel‐wise multiple regression. Low‐frequency noise was removed by 128‐s cutoff high‐pass filtering. No global signal normalization was applied.

For rsfMRI data analysis, a spatial independent component analysis (sICA) approach was used, employing a customized version of the Infomax algorithm running under MATLAB, for the identification of large‐scale networks (Marrelec et al., 2006). Fifty‐five spatial independent components (ICs) were computed on preprocessed images of each individual run. Individual spatial components were thresholded at *z* = 2.

### 2.5 Identification of language and attentional networks

Language network during task‐induced activity was calculated using t‐contrasts SG > TL for each subject and for each session using the framework of the general linear model. Images were corrected for multiple comparisons at the voxel level, with an FWE = 0.05.

Two raters were systematically present for rsfMRI's ICA component identification. However, there was no blind identification and raters were free to exchange on their identification criteria to achieve a consensual choice. Indeed, the primary goal of this study was not to study inter‐raters' variability but to valid the identification of LN on anatomical criteria using MNI template. The arbitrary thresholding of *z* = 2 was chosen for a first visual inspection of ICAs mainly to discriminate noise components and also some easily identifiable ICNs. In a second step, further thresholding at higher *z* values allows identification of peaks of component. As discussed below, identification of these peaks allowed to discriminate LN from other potentially confusing RSNs namely VAN and lFPCN.

Language network at rest (LANGrest) was identified using the same criterion as in a previous study (Ter Minassian et al., 2014): a network presenting activity within subdivisions of the inferior frontal gyrus (IFG) (Bozic, Tyler, Ives, Randall, & Marslen‐Wilson, 2010; Marslen‐Wilson & Tyler, 2007); angular gyrus (ANG) (Vigneau et al., 2006); middle temporal gyrus (MTG) with a peak of activity in its mid‐posterior part (MTG) in the vicinity of superior temporal sulcus (Devlin, Jamison, Matthews, & Gonnerman, 2004; Dronkers & Ogar, 2004); temporal poles (Binder et al., 2011); caudate nucleus (Crosson et al., 2003); cerebellum (Jansen et al., 2005); and dorsomedial prefrontal cortex (Alario, Chainay, Lehericy, & Cohen, 2006). However, we retained the presence of MTG, inferior frontal gyrus, and ANG, either unilaterally or bilaterally, as the main criteria for the identification of LANGrest.

The distinction between LANGrest and the ventral attention network (VAN) was also critical for a proper identification of the language network in rsfMRI, especially in left‐handed patients. Indeed, the VAN presents topographical similarities with the language, with specific activations in the ventrolateral prefrontal cortex, inferior frontal cortex, and temporal gyrus in the right hemisphere in right‐handed subjects (Corbetta, Patel, & Shulman, 2008). The main difference between these two networks lies in the different activation of the inferior parietal lobule. The activity of the parietal lobule in VAN involves the supramarginal gyrus and the temporo‐parietal junction in adults (Corbetta et al., 2008), and also in children (Sylvester et al., 2013), whereas the angular gyrus is preferentially activated in the language network (Vigneau et al., 2006). VAN is also mainly located in the nondominant hemisphere, mirroring the language network. Thus, the presence of a specific activation in the angular gyrus was a major criterion for the identification of LANGrest in left‐handed patients.

Identification of the salience network (SN) was carried out according to the presence of cingulo‐opercular components: dorsal anterior cingulate (dACC), posterior pre‐SMA, and anterior insula/frontal operculum (AIFO) (Farrant & Uddin, 2015; Uddin, Supekar, Ryali, & Menon, 2011).

Considering other RSNs, visual inspection on standard template easily discriminates RSN including primary sensory areas such as visual network and auditory network, the latter being embedded with sensory motor network (Haueisen & Knösche, 2001). There is also no possible confusion with DAN even divided into lateralized subcomponents. LN and DAN may overlap, but the overlapping areas are mainly restricted in inferior frontal gyrus, and DAN presents typical activation of superior intraparietal sulcus, frontal eye field, and lateral occipital cortex involved in motion perception (Vernet, Quentin, Chanes, Mitsumasu, & Valero‐Cabré, 2014). These areas are not components of LN, and their presence is main criteria to discriminate DAN from LN.

A more confusing RSN is indeed the FPC, commonly split into left and right FPC by ICA. Left FPC can be confused with LN when rapidly inspecting elements of ICA. The major criterion is the massive DLPFC and anterior orbitofrontal cortex activity and also inferior parietal gyrus activity for FPC upper of angular gyrus activity (Barredo, Verstynen, & Badre, 2016). Finally, LN and lFPC can be discriminated by the presence of activity in pMTG/superior temporal sulcus activity for LN and more inferior temporal gyrus activity for FPN.

For second‐order group analysis, a paired *t* test was performed between unthresholded MNI normalized task fMRI's contrast maps SG > TL and unthresholded t‐maps of LANGrest. Statistical significance threshold was FWE *p* < .05 corrected for multiple comparisons at the voxel level for the mean language networks identified in task fMRI, rsfMRI, and also for task fMRI > rsfMRI contrast (Figure 2). Statistical significance threshold was defined at FDR *p* < .05 corrected for multiple comparisons at the cluster level using a statistical threshold *p* < .001 uncorrected at the voxel level for the rsfMRI > task fMRI contrast.

Anatomical labels were ascribed to the activation or peak component maxima using the anatomy toolbox for SPM (http://www.fz-juelich.de/inm/inm-1/DE/Forschung/_docs/SPMAnatomyToolbox/SPMAnatomyToolbox_node.html).

Lateralization index (LI) was calculated for each patient from neuroimaging data, using the LI toolbox for SPM (Wilke & Lidzba, 2007; Wilke & Schmithorst, 2006).

### 2.6 Surgical procedures and intraoperative cortical mapping

All patients were operated in awake surgery condition with a cortical and subcortical mapping of language and motor areas. After using the primary motor cortex to set the stimulation intensity threshold, we used the DO 80, the French equivalent of the object denomination task described by Ojemann et al., to identify the area involved in language function (Ojemann, 2003; Ojemann et al., 2008). A speech therapist was present during the surgery to interpret any language disorder and maintain in addition to the test a constant discussion with the patient. Transient language disturbances (aphasic, arrest, paraphasia) were consigned. During surgical removal of the tumor, subcortical stimulation was also used to identify white matter tracts, alternated with ultrasonic hover resection in a back‐and‐forth fashion, as described in a previous study (Delion et al., 2015).

### 2.7 Comparison of cortical mapping to fMRI data

The location of area with speech impairment during cortical mapping was recorded using the neuronavigation and manually reported on the fMRI activation maps. Optical recording shows that ECS maps eloquent areas in a volume of brain tissue up to more than three hundred of mm^3^ and can act on BOLD signal as far as 20 mm of the stimulation site (Borchers, Himmelbach, Logothetis, & Karnath, 2011; Suh, Bahar, Mehta, & Schwartz, 2006). Thus, in our sensitivity analysis we did not consider widespread activation around BOLD peak but the distance around the peaks: We considered the existence of a peak of activity (for task fMRI) or peak of component (for rsfMRI) within 10 mm of the site of ECS.

## 3 RESULTS

### 3.1 Characteristics of the population

Fifty patients were included in this study, 34 men and 16 women (Table 1). The mean age was 49.6 ± 13.5 years (range 18–75 years). Six patients were left‐handed (Table 2). All patients underwent a surgical resection of a brain lesion with intraoperative cortical mapping in awake surgery condition. The histopathological analysis of the brain lesions identified 42 glial tumors (32 high‐grade tumors, 10 low‐grade tumors), 2 metastases, and 6 nontumoral brain lesions: 3 cavernomas, 1 arteriovenous malformation, 1 dysembryoplastic neuroepithelial tumor, and 1 radionecrosis. The mean of self‐evaluated success estimation scores after completion of fMRI acquisitions was 6.4 ± 1.7, and the mean of anxiety scores was 3.4 ± 2.5 on a visual analog scale from 0 to 10. Patients had a statistically significant decrease of the estimated performance to the test and an increased anxiety compared to the success, and anxiety scores of healthy volunteers from a previous study were, respectively, of 7.8 ± 1.3 and 1.7 ± 1.3 (both *p* < .001).

**Table 2:** Laterality indexes

Subjects	Edinburgh	TIA	Rest
1	43	−26	−60
2	8	61	84
3	82	66	70
4	100	3	70
5	82	−41	−57
6	20	3	75
7	−67	−55	82
8	80	22	6
9	82	14	59
10	−80	−28	65
11	100	72	73
12	69	−48	81
13	82	−48	62
14	100	−17	44
15	100	−19	55
16	80	−38	77
17	51	59	49
18	82	25	−30
19	80	−66	91
20	82	32	−82
21	50	−57	81
22	18	9	72
23	5	22	73
24	−100	56	−70
25	−60	10	55
26	100	−20	66
27	82	−14	67
28	80	−9	63
29	80	7	68
30	50	36	86
31	80	−45	86
32	80	26	−81
33	80	−24	78
34	83	21	74
35	60	62	67
36	57	23	−72
37	66	−55	92
38	30	38	42
39	100	13	25
40	100	32	76
41	33	−42	−10
42	−60	1	−87
43	50	46	72
44	25	−12	−48
45	80	12	18
46	100	−55	−4
47	67	24	−12
48	67	−27	−87
49	−60	56	96
50	100	49	−5

Note

All indexes are scaled from −100 (left) to +100 (right). Positive values indicate right handedness on Edinburgh and right hemispheric dominance on TIA and Rest.

Edinburgh: handedness as determined by Edinburgh Handedness Inventory scale; TIA and Rest: Laterality indexes as determined on individual fMRI data of the contrast sentence generation > tone listening and t‐maps of language network isolated at rest, respectively.

Eleven patients had a slight preoperative speech impairment, related to their brain lesion, that recovered sufficiently under medical therapy to allow all patients to perform the preoperative fMRI assessment and the perioperative cortical mapping in awake surgery condition.

### 3.2 Identification of language networks and laterality indexes

**Figure 1:**
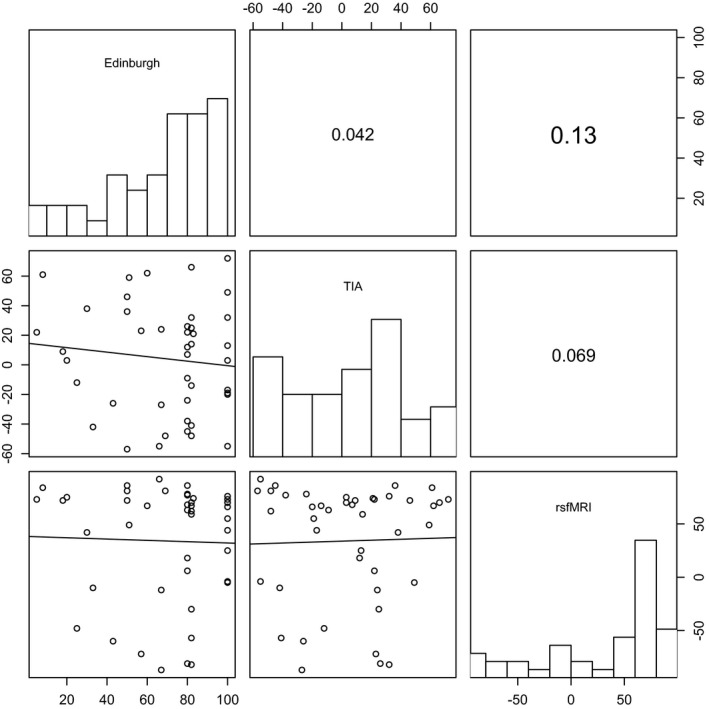
fMRI rendering of mean activation peaks in identified language networks in TIA and rsfMRI. task fMRI FDR < 0.05 and *t* = 5,617, cluster‐size threshold five voxels; rsfMRI FDR < 0.05 and *t* = 5,617, cluster‐size threshold five voxels; task fMRI > rsfMRI contrast, *p* < .001 corrected at the cluster level; rsfMRI > task fMRI contrast, FDR < 0.05 and *t* = 5,617, cluster‐size threshold five voxels

The contrast SG > TL identified significant clusters (Figure 1a, Table 3). In eight patients, we were unable to identify significant clusters with the task fMRI paradigm. In rsfMRI, the language network, along with other networks including the left fronto‐parietal control network, the VAN, the salience network, and the default mode network, was identified in all patients.

**Table 3:** Mean activation peaks of the language network identified in task fMRI

Location	*k*	*t*‐score	Cytoarchitectonic location	MNI coordinates		
				*x*	*y*	*z*
R Insula Lobe	2,051	5.15		48	12	−2
R IFG (p. Opercularis)		5.14		45	14	4
R IFG (p. Orbitalis)		5.08		54	30	−9
R Rolandic Operculum		4.32		51	9	3
L Posterior Medial Frontal	1,632	4.56		−9	17	60
L ACC		4.45		−3	27	28
L MCC		4.28		−11	18	33
L Superior Medial Gyrus		4.21		−6	26	46
L IFG (p. Orbitalis)	1,545	5.46		−41	23	−14
L Insula Lobe		5.01		−38	18	−9
L Temporal Pole		4.78		−47	18	−17
R Cerebellum (Crus 1)	1,487	4.89	R Lobule VIIa crusI (Hem)	39	−63	−27
L Cerebellum (VI)	1,375	4.48	L Area FG2	−39	−67	−21
L Cerebellum (Crus 1)		4.39	L Lobule VIIa crusI (Hem)	−47	−66	−30
L Inferior Temporal Gyrus		4.38		−59	−61	−15
L Fusiform Gyrus		4.23	L Area FG2	−44	−66	−20
R Cerebellum (VIII)	370	4.22		33	−60	−50
Cerebellar Vermis (7)	331	4.44	R Lobule VI (Verm)	5	−78	−20
R Cerebellum (Crus 1)		3.80	R Lobule VI (Verm)	11	−82	−21
L Inferior Parietal Lobule	250	4.45	L Area PF (IPL)	−54	−37	46
L Precentral Gyrus	248	4.47		−39	−3	34
L Cerebellum (VIII)	248	4.33	L Lobule VIIb (Hem)	−35	−60	−51
R Caudate Nucleus	171	4.97		18	8	19
L Superior Frontal Gyrus	170	4.59		−23	44	21
L Middle Frontal Gyrus		4.49		−20	44	15
R Middle Temporal Gyrus	141	4.17		66	−40	−3
L Supramarginal Gyrus	134	4.30	L Area PF (IPL)	−65	−36	33
R IFG (p. Opercularis)	132	4.20	R Area 44	57	8	13
R Precentral Gyrus		3.96	R Area 44	60	8	19
R Middle Frontal Gyrus	100	4.02		37	−1	52
L Middle Orbital Gyrus	78	3.96		−45	45	−3
L IFG (p. Orbitalis)		3.88		−45	38	−6
L Thalamus	77	4.42	L Thal: Temporal	−6	−7	10
R Middle Frontal Gyrus	55	4.02		42	41	25
R IFG (p. Triangularis)		3.74		46	36	25
R Thalamus	46	3.66	R Thal: Prefrontal	18	−10	16
R Caudate Nucleus		3.55		15	−9	21
L Caudate Nucleus	42	3.82		−12	−4	18
L Posterior Medial Frontal	23	3.96		0	5	66

Note

The significant local peak maxima were obtained using a one‐sample *t* test corrected for multiple comparisons under a threshold of *p* .001 at the cluster level, cluster‐size threshold 39 voxels; anatomical labels were derived from anatomy toolbox for SPM; *k* = cluster extend in voxels, in case of multiple peaks in the same anatomic area of a cluster, only the maximal peak is presented for this anatomic area; *x*, *y*, and *z* = original SPM coordinates in the MNI space in millimeters.

Abbreviations: fMRI, functional magnetic resonance imaging; MNI, Montreal Neurological Institute.

The mean image of significant clusters identified with the task fMRI paradigm showed significant activations in brain area classically involved in language: the left inferior frontal gyrus, the posterior medial frontal gyrus, both temporal lobes, left temporal pole, the left inferior parietal lobule, and the right cerebellar hemisphere. We also identified in task fMRI elements of the salience network in the language network, including activity in the anterior insula, the frontal operculum, and the dorsal anterior cingulate cortex.

The mean image of language networks identified in rsfMRI showed significant bilateral activity of the angular gyri, MTG, temporal poles, the inferior frontal gyri, the posterior frontal gyri, and the right cerebellar hemisphere with a predominant activity lateralized on the left (Figure 1b, Table 4). The highest activation peak was identified in left MTG.

**Table 4:** Mean activation peaks of the language network identified in rsfMRI

Location	*k*	*t*‐score	Cytoarchitectonic location	MNI coordinates		
				*x*	*y*	*z*
L Middle Temporal Gyrus	8,333	10.29		−63	−28	−9
L Supramarginal Gyrus		10.27	L Area PFm (IPL)	−60	−52	28
L Angular Gyrus		10.25	L Area PGa (IPL)	−54	−55	30
L IFG (p. Orbitalis)	2,262	8.41		−47	27	−9
L IFG (p. Triangularis)		7.39	L Area 45	−54	21	6
L Posterior Medial Frontal	1,064	6.76		−6	17	64
L Superior Medial Gyrus		6.54		−6	24	63
L Superior Frontal Gyrus		6.35		−14	23	60
L Posterior Medial Frontal		6.30		−5	24	58
L Middle Frontal Gyrus	946	7.93		−44	9	51
L Precentral Gyrus		7.82		−41	6	49
R Cerebellum (Crus 1)	499	6.26	R Lobule VIIa crusI (Hem)	24	−76	−30
R Cerebellum (Crus 2)		5.70	R Lobule VIIa crusII (Hem)	24	−84	−42
R IFG (p. Orbitalis)	297	5.96		51	30	−11
R Middle Temporal Gyrus	225	6.12		62	−30	−11
L Temporal Pole	211	6.82		−51	21	−11
R Superior Frontal Gyrus	78	5.77		15	59	24
R Superior Medial Gyrus		5.71		14	59	30
R Angular Gyrus	63	5.73	R Area PGa (IPL)	57	−55	36
L Precuneus	46	5.70		−6	−49	39
L Superior Medial Gyrus	27	5.65		−8	45	45
R Superior Medial Gyrus	15	5.51		9	29	58
L IFG (p. Triangularis)	15	5.58		−50	15	30
L Superior Medial Gyrus	10	5.44		−6	51	30

Note

The significant local peak maxima were obtained using a FWE *p* < .05 at the voxel level, cluster‐size threshold five voxels; anatomical labels were derived from anatomy toolbox for SPM; *k* = cluster extend in voxels, in case of multiple peaks in the same anatomic area of a cluster, only the maximal peak is presented for this anatomic area; *x*, *y*, and *z* = original SPM coordinates in the MNI space in millimeters.

Abbreviations: MNI, Montreal Neurological Institute; rsfMRI, resting‐state fMRI.

**Figure 2:**
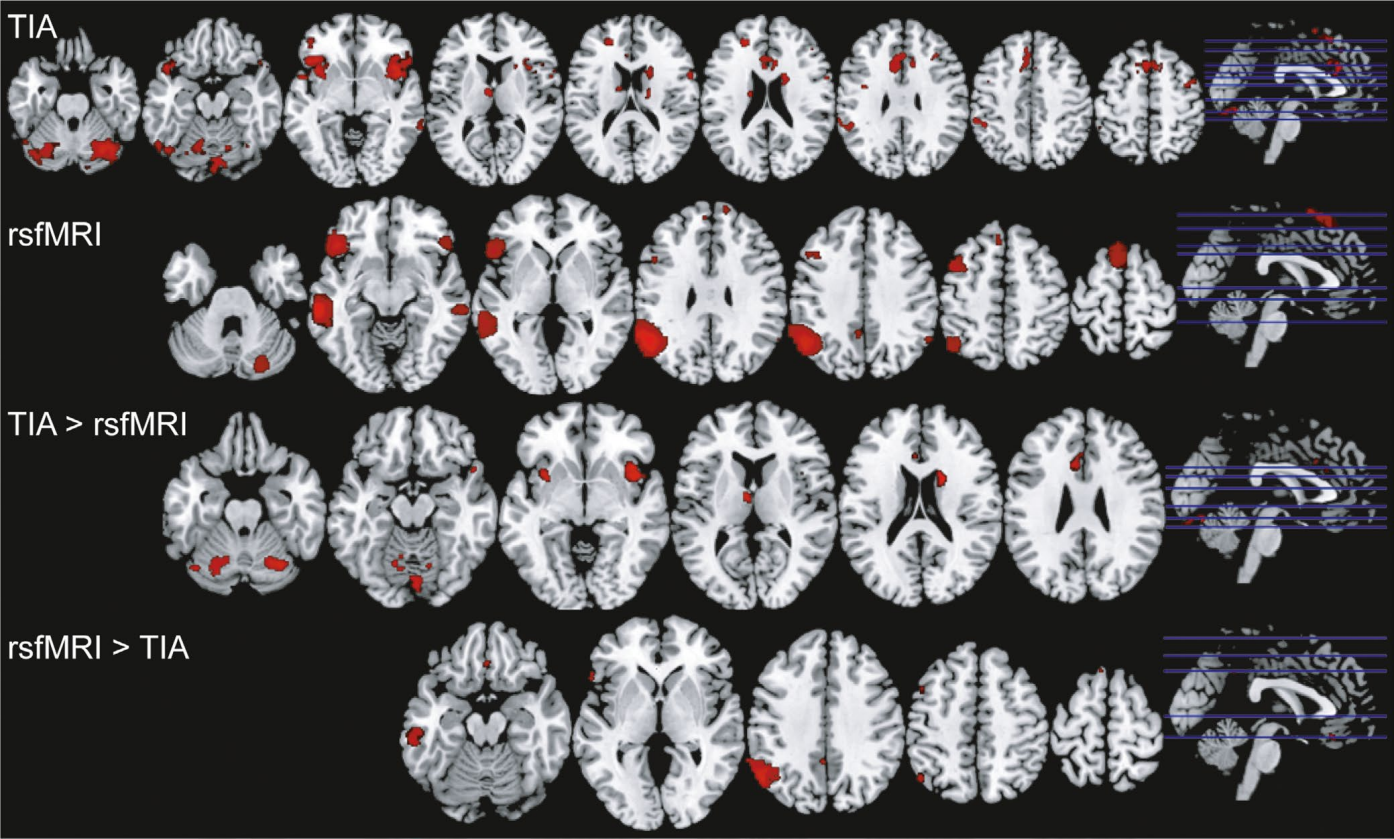
Correlation between laterality indexes of the Edinburgh Handedness Inventory and the language networks identified in task‐induced activity and resting‐state MRI in right‐handed patients. For each laterality index, the histogram is displayed in diagonal, the correlation coefficients between the different pairs in the upper right and the scatter plot with the fitted linear regression model in the lower left

Among the 44 right‐handed patients, no correlation was found between Edinburgh Handedness Inventory Score, task fMRI, and rsfMRI laterality indexes (Figure 2, Table 2). In the left‐handed population subgroup of six patients, the Edinburg score was −0.71 ± 0.16 and the laterality indexes of identified language in task‐based fMRI and rsfMRI showed a predominant activation in the right hemisphere in, respectively, 3/6 and 4/6 patients.

### 3.3 Comparison of language networks identified with task‐based fMRI and resting‐state fMRI

The paired *t* test used to calculate the main effect of task fMRI > rsfMRI showed significant higher signal in several brain regions, including areas previously described to be a part of the salience network: the dorsal anterior cingulate cortex and the right AIFO (Figure 1c and Table 5).

**Table 5:** Paired *t* test, greater activity in task fMRI compared to rsfMRI

Location	*k*	*t*‐score	Cytoarchitectonic location	MNI coordinates		
				*x*	*y*	*z*
R Insula Lobe	685	4.77		42	12	−8
R IFG (p. Opercularis)		4.32		43	12	4
R Rolandic Operculum		4.19		49	8	3
R Temporal Pole		3.97		51	14	−12
R Cerebellum (VI)	450	4.34	R Lobule VI (Hem)	23	−60	−30
R Cerebellum (Crus 1)		3.52	R Lobule VI (Hem)	30	−60	−33
L Cerebellum (VI)	442	3.98	L Lobule VI (Hem)	−15	−63	−17
L ACC	246	4.30		−11	17	30
L MCC		3.85		−9	11	36
L Cerebellum (Crus 1)	211	4.07	L Area FG2	−41	−66	−21
Cerebellar Vermis (7)	188	4.12	R Lobule VI (Verm)	5	−78	−20
Cerebellar Vermis (6)		3.96	L Lobule VI (Verm)	0	−76	−15
R Cerebellum (VI)		3.51	R Area hOc2 [V2]	12	−81	−17
R Caudate Nucleus	92	4.90		18	8	19
L Thalamus	59	4.40	L Thal: Temporal	−6	−7	10

Note

The significant local peak maxima were obtained under a threshold of *p* .001 corrected for multiple comparisons at the cluster level, cluster‐size threshold 59 voxels; anatomical labels were derived from anatomy toolbox for SPM; *k* = cluster extend in voxels, in case of multiple peaks in the same anatomic area of a cluster, only the maximal peak is presented for this anatomic area; *x*, *y*, and *z* = original SPM coordinates in the MNI space in millimeters.

Abbreviations: fMRI, functional magnetic resonance imaging; MNI, Montreal Neurological Institute; rsfMRI, resting‐state fMRI.

The inverse contrast rsfMRI > task fMRI identified several higher activated areas, including the left angular gyrus, temporal pole, middle temporal gyrus, and inferior frontal gyrus, key elements of the language network (Figure 1d and Table 6).

**Table 6:** Paired *t* test, greater activity in rsfMRI compared to task fMRI

Location	*k*	*t*‐score	Cytoarchitectonic location	MNI coordinates		
				*x*	*y*	*z*
L Supramarginal Gyrus	2,666	10.14	L Area PFm (IPL)	−60	−52	28
L Angular Gyrus		10.02	L Area PGa (IPL)	−53	−61	34
L Angular Gyrus		7.03	L Area PFm (IPL)	−47	−61	51
L Middle Temporal Gyrus	442	8.90		−62	−31	−14
L IFG (p. Triangularis)	29	6.73	L Area 45	−57	21	−2
L Middle Frontal Gyrus	19	6.67		−47	9	52
L Rectal Gyrus	18	6.85		2	35	−18
L Precuneus	10	6.78		−3	−48	37
L Temporal Pole	9	6.71		−50	21	−11
L Middle Temporal Gyrus	8	6.60		−59	−46	−5
L Posterior Medial Frontal	5	6.77	L Thal: Temporal	−6	27	66

Note

The significant local peak maxima were obtained under a threshold of FWE *p* < .05 corrected for multiple comparisons, *t* = 5.617, cluster‐size threshold five voxels; anatomical labels were derived from anatomy toolbox for SPM; *k* = cluster extend in voxels, in case of multiple peaks in the same anatomic area of a cluster, only the maximal peak is presented for this anatomic area; *x*, *y*, and *z* = original SPM coordinates in the MNI space in millimeters.

Abbreviations: fMRI, functional magnetic resonance imaging; MNI, Montreal Neurological Institute; rsfMRI, resting‐state fMRI.

### 3.4 Differences in brain activations between perioperative language cortical mapping with both resting‐state fMRI and task‐based fMRI

All patients had a perioperative cortical mapping, and brain language areas were identified perioperatively using cortical mapping in 32 of them. The individual brain activations of language networks identified in task fMRI and rsfMRI compared to the perioperative cortical mapping are presented in Table 7. The rsfMRI had a sensitivity of 100% for the identification of eloquent brain language area during surgery, whereas the sensitivity of task fMRI analysis was 65.6%. Furthermore, rsfMRI successfully identified functional brain language areas in four patients where task fMRI did not succeed to identify any significant cluster (patients 3, 15, 16, and 28). Among the 18 patients with a negative cortical mapping, 14 of them had brain language identified in task‐based fMRI exposed through the craniotomy during the surgical procedure and 15 had brain language areas identified in rsfMRI.

**Table 7:** Main findings obtained on fMRI data and by electrical cortical mapping during awake craniotomy. Anatomic areas in bold are eloquent areas identified by rsfMRI but not by task fMRI

Subjects	Task fMRI							rsfMRI							CM
	MTG	ANG	TP	IFG	SMA/pre‐SMA	dACC	AIFO	MTG	ANG	TP	IFG	SMA/pre‐SMA	dACC	AIFO	Site of induced aphasia or paraphasia
1	Bi	Bi	Bi	Bi	Bi	Bi	Bi	Bi	Bi	No	Bi	No	Bi	No	No induced language disturbance
2	Left	Left	No	Left	No	No	No	Left	Left	Left	Left	No	No	No	No induced language disturbance
3	No	No	No	No	No	No	No	**Left**	Left	No	Left	Bi	No	No	**Left MTG**
4	No	Bi	No	No	No	No	No	**Left**	Bi	No	Left	Bi	No	No	Left ANG, **left MTG**
5	Bi	Left	No	Left	Bi	Bi	Bi	Bi	Bi	Left	Bi	No	Bi	Left	Left IFG
6	Bi	Left	No	Bi	Bi	No	Bi	Bi	Left	No	Bi	Left	Left	No	Left IFG
7	Right	No	No	No	No	Bi	No	Bi	Left	No	**Left**	No	No	No	**Left IFG**
8	Left	Left	No	Left	No	Bi	Right	Left	Left	No	Bi	No	No	Right	Left IFG
9	No	Bi	Left	No	No	No	No	Bi	Bi	Right	Bi	No	No	No	No induced language disturbance
10	Bi	Bi	Bi	Left	No	Bi	Left	Bi	No	No	Bi	No	No	Right	No induced language disturbance
11	Bi	Left	No	Bi	Bi	No	Bi	Bi	Right	Right	Bi	No	Bi	No	Left MFG
12	Bi	Left	No	Bi	Bi	Bi	Right	Bi	Bi	No	Bi	No	No	No	Left IFG
13	Left	Bi	No	Left	No	Bi	Bi	Bi	Bi	No	Bi	No	No	Left	Left IFG
14	Bi	Bi	Bi	Bi	Bi	Bi	Bi	Bi	Bi	Right	Bi	No	No	No	No induced language disturbance
15	No	No	No	No	No	No	No	Bi	Bi	Right	**Bi**	No	No	No	**Left IFG**
16	No	No	No	No	No	No	No	Bi	Right	Right	**Bi**	No	Left	No	**Left IFG**
17	Bi	Left	No	Left	Bi	Bi	Bi	Bi	Right	No	Bi	No	Left	No	Left IFG
18	No	Left	No	No	No	No	No	Bi	Left	No	Bi	No	Left	No	Left ANG
19	Right	Bi	No	Bi	No	Right	Right	Left	Left	No	Bi	No	No	Right	Left IFG
20	Bi	Right	Bi	Bi	Bi	Bi	Bi	Bi	Right	No	Bi	No	Right	No	Left IFG
21	Bi	Bi	No	Bi	Bi	Bi	Bi	Left	Bi	No	Bi	No	No	No	Left ANG
22	Left	Left	No	No	No	No	No	Bi	Bi	No	Bi	No	No	No	Left MTG
23	No	No	No	No	No	No	No	Bi	Bi	No	Bi	No	No	No	No induced language disturbance
24	No	No	No	No	Left	No	No	Bi	Bi	Left	Bi	No	No	No	No induced language disturbance
25	Bi	Bi	Left	Bi	Bi	Bi	Bi	Bi	Bi	No	Right	Right	No	No	**Right IFG**
26	Left	No	Left	Bi	No	No	Bi	Left	Right	Bi	Bi	No	Left	No	No induced language disturbance
27	No	No	No	No	No	Bi	No	Bi	Left	Left	Left	No	No	No	Left SMA/pre‐SMA
28	No	No	No	No	No	No	No	Bi	Left	No	Bi	No	Left	No	**Left superior frontal gyrus**
29	No	Bi	No	No	No	No	No	Bi	Bi	Left	Bi	No	No	No	No induced language disturbance
30	Right	No	No	No	No	No	No	Bi	No	Right	**Bi**	No	No	No	**Left IFG**
31	Bi	Bi	No	Left	Bi	No	Bi	Bi	Left	No	Bi	No	No	No	Left MTG
32	Right	No	No	No	No	Bi	No	**Bi**	Bi	Right	Bi	No	No	No	**Left MTG**
33	No	No	No	No	No	No	No	Bi	Bi	Right	Bi	No	No	No	No induced language disturbance
34	Bi	Bi	No	Bi	Bi	Bi	Bi	Right	Right	Right	Bi	No	No	No	No induced language disturbance
35	Bi	Left	No	Right	No	Bi	Right	Bi	Left	No	Bi	No	No	No	No induced language disturbance
36	Bi	Bi	Left	Bi	Bi	Left	Bi	Bi	Bi	Left	Bi	No	No	No	Left ANG
37	Bi	Bi	No	Left	Left	No	Left	Bi	Left	No	Bi	No	No	No	Left MTG
38	No	No	No	No	No	No	No	Bi	Left	No	Bi	No	No	No	No induced language disturbance
39	Right	Bi	No	Bi	Bi	No	Bi	Bi	Right	Right	Left	No	Bi	No	No induced language disturbance
40	Left	Bi	No	Bi	Bi	No	No	Bi	Left	Left	Bi	No	Bi	No	Left IFG
41	Left	Bi	No	Bi	No	No	No	Bi	Left	Right	Bi	No	No	No	No induced language disturbance
42	Bi	Bi	No	Bi	Bi	No	Bi	Bi	Bi	No	Right	Left	No	No	**Left superior frontal junction**
43	Left	Left	Left	Bi	No	Bi	Left	Left	Left	Right	Left	No	No	Right	No induced language disturbance
44	Left	Bi	Bi	Left	Bi	No	Bi	Bi	Left	Left	Left	Left	No	Left	Left middle temporal gyrus
45	Left	Bi	Bi	Bi	Bi	No	Bi	Bi	Bi	Bi	Left	Left	No	Left	Posterior part of left middle frontal gyrus
46	No	Bi	Bi	Bi	Bi	No	Bi	Bi	Bi	Right	Bi	Right	Left	No	**Left middle temporal gyrus**
47	No	Bi	Bi	Bi	Bi	Bi	Bi	Left	Bi	Bi	Left	Bi	Bi	Right	No induced language disturbance
48	No	Bi	Bi	Bi	Bi	No	Right	Bi	Bi	Bi	Left	No	Bi	No	Middle part of left inferior frontal gyrus
49	Left	Bi	Bi	Bi	Bi	Bi	Bi	Left	Left	No	Left	Bi	No	Left	No induced language disturbance
50	Left	Bi	Bi	BI	Bi	Left	Bi	Bi	Bi	Bi	Bi	Left	No	No	No induced language disturbance

Abbreviations: AIFO, anterior insula–frontal operculum; ANG, angular gyrus; Bi, bilateral; CM, cortical mapping; dACC, dorsal anterior cingulate cortex; IFG, inferior frontal gyrus; fMRI, functional magnetic resonance imaging; rsfMRI, resting‐state fMRI; Rest, resting‐state fMRI analyzed by spatial independent components analysis; SMA/pre‐SMA, supplementary and presupplementary motor area; MTG, posterior middle temporal gyrus; TIA (GLM), task‐induced activity analyzed by general linear model; TP, temporal pole.

## 4 DISCUSSION

### 4.1 Identification of the language network in rsfMRI and task fMRI

This study has shown the possibility to isolate the language network in resting‐state fMRI, even in patients with atypical lateralization or brain lesions.

In healthy volunteers, LANGrest was identified as a left lateralized network in right‐handed subjects. Indeed, 82%–96% of right‐handed individuals use their left hemisphere for language processing (Knecht, Deppe, et al., 2000; Knecht, Dräger, et al., 2000; Springer et al., 1999). This criterion remains true in left‐handed people but is weaker. Moreover, the incidence of right hemisphere dominance is linearly correlated with the degree of handedness on EHI, ranging from 4% when EHI = 100%–27% when EHI = −100 (Knecht, Deppe, et al., 2000). As the correlation between LI indexes from the Edinburgh Handedness Inventory Score, task fMRI, and rsfMRI was poor, little emphasis has been put on LI for the identification of LANGrest but the study has been much stricter regarding anatomic criteria. As exposed previously, VAN presents similarities in the right hemisphere with LANGrest notably in the IFG and temporal gyrus. Thus, we suggest that when attempting to identify LANGrest by sICA, identification of VAN should also be performed in such a way to discriminate these two networks by their different activity in the inferior parietal lobule, with the involvement of the angular gyrus for language network (Vigneau et al., 2006) and the supramarginal gyrus for VAN (Corbetta et al., 2008).

For a methodological standpoint, we acknowledge that the comparison of task fMRI and rsfMRI using thresholded t‐maps may be subject to discussion, as the two techniques are based on different statistical methodologies. Both techniques are derived from the BOLD signal in fMRI but differ from their neurophysiological basis, the task‐based fMRI relies on the specific activation of brain areas during a language task, whereas rest fMRI is based on BOLD signal oscillation synchronization between distant brain areas. We confront both techniques to the gold standard, the perioperative electric cortical stimulation in awake surgery not to identify which technique is the best from a methodological point of view, but to find the one that is the most relevant and sensitive for the presurgical mapping of language functional areas with the aim to preserve patient neurological function. Thus, in this perspective, the direct comparison of both techniques appears relevant.

### 4.2 Salience network and language

The literature is quite confusing as to the definition of the VAN and the SN, which may be explained by differences in nomenclature and methodologies. VAN was first identified in rsfMRI by Fox, Corbetta, Snyder, Vincent, and Raichle (2006) and described as a RSN correlated to a region of interest that has since been shown to be part of SN (Uddin et al., 2011). Following this first description, Srhidaran et al. indiscriminately referred to VAN and SN as the same network (Sridharan, Levitin, Chafe, Berger, & Menon, 2007; Sridharan, Levitin, & Menon, 2008). This was also put forth in one of our previous study on language network connectivity and in an important paper on neurolinguistics (Ter Minassian et al., 2014; Vaden et al., 2013). Recent work on connectivity has shown that VAN and SN are separate networks: the temporo‐parietal junction being a key cluster of VAN and the dorsal part of the anterior cingulate cortex being a key cluster of the latter (Farrant & Uddin, 2015). Current findings of distinct networks identified by sICA linked to these areas are in line with the results of Farrant et al., and in our study, VAN and SN are distinct spatial components.

In this study, a coactivation of the main nodes of SN together with the language network was observed during task fMRI. The presence of SN nodes in task fMRI may be linked to the experimental block design of the task‐based acquisition and is not surprising according to the difficult acoustic condition inherent to MRI. Indeed, coactivation of the SN during a linguistic task supports word identification in difficult acoustic conditions (Vaden et al., 2013). The presence of SN nodes in fMRI may also explain the stronger right lateralization observed in language network in fMRI compared to LANGrest, since the SN is slightly right lateralized. Also, the choice of a high number of ICA generated from the rsfMRI acquisition may play a role and fragment the language network through several ICAs. However, we think that this was not the case in our study as the chosen number of generated ICA is in accordance with the literature and identified the main activation peaks described in the literature (Geranmayeh, Wise, Mehta, & Leech, 2014).

### 4.3 Identification of semantic areas in the language network in rsfMRI

The second‐level analysis showed significant activation peaks in both angular gyri and temporal poles in rsfMRI, known to be involved in semantic processing (Binder, Desai, Graves, & Conant, 2009; Binder et al., 2011; Vigneau et al., 2006). It has been emphasized that task fMRI, obtained by contrasting a linguistic task to a low‐level baseline (tones), has poor sensibility in detecting semantic areas. Indeed, mind wandering, which also activates semantic processing, is likely to occur during a low‐level baseline. With the semantic system being active during the linguistic task and baseline, it is no more visible in the contrast image between these two conditions (Binder et al., 2011). A contrast using a task requiring a high level of attentional control has been recommended to identify the semantic network (Binder et al., 2011) but may also be difficult to perform by patients. Thus, rsfMRI, detecting systematically semantic areas, appears as a good alternative to a task requiring a high level of attentional control.

### 4.4 rsfMRI versus task‐based fMRI for the preoperative identification of brain functional language areas

Resting‐state fMRI detected all eloquent areas identified preoperatively with CM, compared to the classical task‐based paradigm that had a sensitivity of 65.6%. This illustrates the interest of rsfMRI for the presurgical mapping of brain language area. However, it is necessary to fulfill certain conditions to reach a high sensitivity in the preoperative language mapping using rsfMRI. First, we do not make aphasic patients talk: All patients with a preoperative language disturbance that did not improve sufficiently prior to surgery with medical treatment to be eligible for cortical mapping were excluded from this study. Furthermore, as detailed above, we carefully identified the language network in rsfMRI, especially by differentiating it from the VAN and also from lFPCN. In this study, it was indeed not feasible to assess the specificity of the technique since it requires the cortical mapping of the whole‐brain surface during the surgery to identify false positives in fMRI.

Resting‐state fMRI has the advantage to overcome the limitations of task‐based fMRI in terms of task performance requirements and the spontaneous fMRI oscillation recorded in rsfMRI persist in sleep or anesthesia condition (Fukunaga et al., 2006; Vincent et al., 2007). This allows the inclusion of patients unable to perform the functional task, stressed patients, and even young children. Another advantage is the possibility to identify many different networks in one data acquisition, reducing acquisition time when several functional networks are studied. One of the main difficulties of this method is the determination of the total number of components (TNC) to be used, which may lead to suboptimal decompositions with the merging of multiple networks in case of low TNC, or the fragmentation of a functional network into multiple components in case of high TNC (Li, Adali, & Calhoun, 2007; Sair et al., 2016). Our choice to analyze 55 ICs among all patients was based on a previous work and appeared to be a good compromise (Geranmayeh et al., 2014). The identification of functional networks using traditional visual inspection is time‐consuming, experience‐dependent, and sometimes biased. These errors can alter the final result (Greicius, 2008). Furthermore, due to neurovascular uncoupling in the vicinity of the tumor, it could be a loss of BOLD signal, which may reduce the sensitivity of our analysis (Agarwal, Sair, Airan, et al., 2016; Agarwal, Sair, Yahyavi‐Firouz‐Abadi, Airan, & Pillai, 2016). However, rsfMRI was able in our study to isolate functional brain area related to language in four patients without statistically significant language network in task fMRI and had a sensitivity of 100% compared to the gold standard: Such a loss of neurovascular uncoupling appears unlikely in our rsfMRI analysis.

There are few studies in the literature on presurgical motor and language mapping by rsfMRI. They mostly consisted of technical notes or case reports of a few patients (Delion et al., 2015; Kamran et al., 2014; Lee, Smyser, & Shimony, 2013; Shimony et al., 2009; Zhang et al., 2009). The only studies reporting the comparison of task fMRI and rsfMRI to direct intraoperative stimulation were a series of 13 patients from Mitchell et al., where rsfMRI showed a good sensibility in the identification of motor and language functional brain areas (Mitchell et al., 2013). Other multichannel modalities like the high‐density electroencephalogram or magnetoencephalography have also been used to identify language network areas (Kambara et al., 2018; Tierney et al., 2018).

The next step in the development of our rsfMRI analysis will be to automate the network detection neural learning algorithm in rsfMRI to minimize the bias associated with the visual selection of the language network.

### 4.5 Limitations of the study

Our original work on the comparison of task and rest fMRI to perioperative mapping for the identification of language network suffers for several limitation. First, the choice of the denomination task for the perioperative mapping may be subject to question as it does not solicit all brain areas involved in language. To avoid this issue, all patients were also tested in spontaneous language by an experienced speech therapist.

Also, recent guideline for presurgical language mapping recommends the performance of at least a verbal fluency and a lexical/semantic task such as noun–verb matching (Zacà, Jarso, & Pillai, 2013). The paradigm we used here consists of a kind of noun–verb association as the patient was instructed to covertly match with the noun a short contextually related sentence. Indeed, a sentence always includes a verb. This was done because for some patients, this task was easier to perform than strict but more abstract noun–verb matching. As shown in Supporting information, in healthy volunteers, this task is able to induce robust activation within main linguistic areas including temporal poles involved in semantics. We hypothesize that some psychological factor such as stress is responsible of poor performance in our patients leading to poor activations when performing the proposed linguistic tasks. An argument in favor of this hypothesis is the fact that patients reported a lower estimated success and a higher anxiety than healthy volunteers.

As we discussed and as described in previous studies, the low cognitive level of our control block could have resulted in higher activity of semantic areas during the control block and hence to weaker semantic contrast specially in left angular gyrus (Binder et al., 2009). Overall, this could have affected the sensitivity of task MRI compared to rsfMRI. However, our results indicate that when patients are poorly performing task fMRI, rsfMRI allows identification of main nodes of LN.

We were not able to perform a specificity analysis and compare task‐based and rest fMRI. With our perioperative setting for the evaluation of brain language areas, it was difficult to assess the specificity of the fMRI techniques that studies the whole‐brain activation in the limited brain surface offered to examination by craniotomy. For example, the dorsal anterior cingulate cortex and the anterior insula–frontal operculum, commonly activated in task‐based fMRI, as we discussed, are rarely tested perioperatively using electric cortical stimulation due to their deep location, usually away for tumor locations eligible to awake surgery procedures. However, the activation volume of rsfMRI language network was larger to the activation volume in task fMRI (13,880 activated voxels vs. 10,766), suggesting a supposed higher specificity of task‐based fMRI that may explain conversely the higher sensitivity of rsfMRI.

Rest fMRI identified brain language networks in the 18 patients without language network retrieved through cortical stimulation, whereas brain language areas were identified in 15 of these patients using task fMRI. After careful review of the craniotomies and the exposed brain surface available to electric cortical stimulation, 14 patients with negative cortical mapping had brain language areas identified in task‐based fMRI exposed by the craniotomy and 15 in rsfMRI. We should also consider the fact that there is also false positive in fMRI cartography. For example, activation of temporal poles in language network is a common feature but the occurrence of language impairment after temporal pole resection is extremely rare when performing a temporal lobectomy using as posterior limit the Labbé vein. We identified left temporal in more than 50% of our patients by rsfMRI. These two points seem to indicate a lesser specificity of both fMRI techniques compared to electric cortical stimulation. Future studies, specifically designed, could confirm the lesser specificity of both fMRI modalities compared to cortical mapping.

Indeed, we detected some peaks of BOLD signal on both task and rsfMRI without language disruption by ECS. However, we never observed language disruption by ECS without a peak BOLD signal on LN isolated by sICA in the immediate vicinity.

## 5 CONCLUSION

In our study, resting‐state fMRI for presurgical language mapping is a technique easy to implement, allowing the identification of functional brain language area with a greater sensitivity than the task‐based fMRI, at the cost of some precautions and a lower specificity. Resting‐state fMRI may become a tool of choice for the presurgical identification of brain language areas, improving the presurgical planning for brain tumor operated in awake surgery condition. Further study is required to compare both the sensitivity and the specificity of the two methods and to evaluate the clinical value of rsfMRI as an alternative tool for the presurgical identification of brain language areas.

## CONFLICT OF INTEREST

None declared.

## Data Availability

Research data are not shared.
