# Supplementary figures and images for: Resting‐state functional magnetic resonance imaging versus task‐based activity for language mapping and correlation with perioperative cortical mapping
Source: Brain Behav. 2019 Sep 30;9(10):e01362. doi: 10.1002/brb3.1362 (PMC6790308; doi:10.1002/brb3.1362)

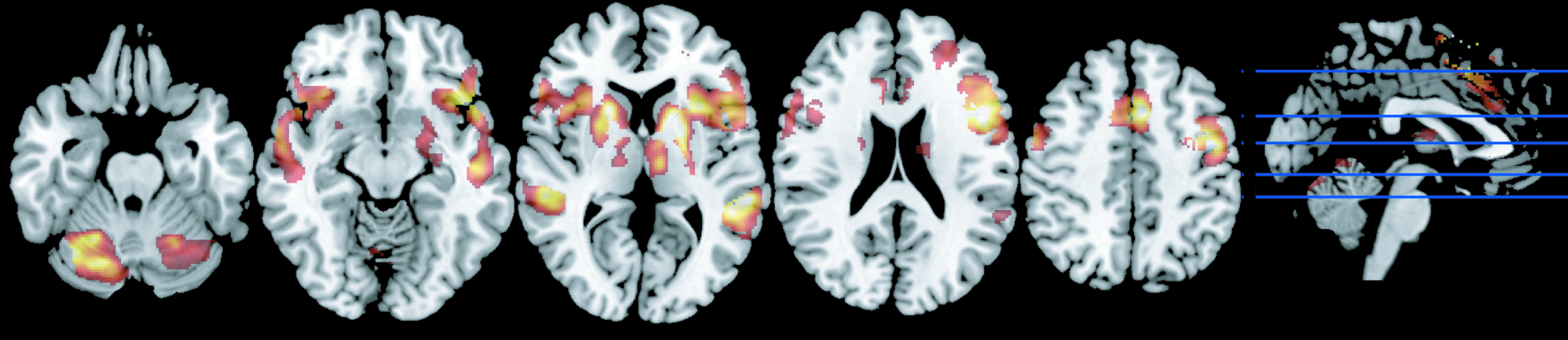

Supplement: Supplementary file 1 [file BRB3-9-e01362-s001.tif]
